# Exome sequencing identifies pathogenic variants of *VPS13B* in a patient with familial 16p11.2 duplication

**DOI:** 10.1186/s12881-016-0340-0

**Published:** 2016-11-10

**Authors:** Jila Dastan, Chieko Chijiwa, Flamingo Tang, Sally Martell, Ying Qiao, Evica Rajcan-Separovic, M. E. Suzanne Lewis

**Affiliations:** 1 Department of Medical Genetics, Children’s and Women’s Health Center of BC and Children’s and Women’s Health Center of BC and Univeristy of British Columbia, Vancouver, BC V6H 3N1 Canada; 2 Department of Pathology (Cytogenetics), Children’s and Women’s Hopsital of BC and Children’s and Women’s Health Center of BC and Univeristy of British Columbia, Vancouver, BC V5Z 4H4 Canada; 3 BC Children’s Hospital Research Institute, Vancouver, BC V6H 3N1 Canada

**Keywords:** 16p11.2 duplication, Cohen syndrome, Neuro-developmental disorders, Variable expressivity, Whole exome sequencing, Case report

## Abstract

**Background:**

The recurrent microduplication of 16p11.2 (dup16p11.2) is associated with a broad spectrum of neurodevelopmental disorders (NDD) confounded by incomplete penetrance and variable expressivity. This inter- and intra-familial clinical variability highlights the importance of personalized genetic counselling in individuals at-risk.

**Case presentation:**

In this study, we performed whole exome sequencing (WES) to look for other genomic alterations that could explain the clinical variability in a family with a boy presenting with NDD who inherited the dup16p11.2 from his apparently healthy mother. We identified novel splicing variants of *VPS13B* (8q22.2) in the proband with compound heterozygous inheritance. Two *VPS13B* mutations abolished the canonical splice sites resulting in low RNA expression in transformed lymphoblasts of the proband. *VPS13B* mutation causes Cohen syndrome (CS) consistent with the proband’s phenotype (intellectual disability (ID), microcephaly, facial gestalt, retinal dystrophy, joint hypermobility and neutropenia).

The new diagnosis of CS has important health implication for the proband, provides the opportunity for more meaningful and accurate genetic counselling for the family; and underscores the importance of longitudinally following patients for evolving phenotypic features.

**Conclusions:**

This is the first report of a co-occurrence of pathogenic variants with familial dup16p11.2. Our finding suggests that the variable expressivity among carriers of rare putatively pathogenic CNVs such as dup16p11.2 warrants further study by WES and individualized genetic counselling of families with such CNVs.

**Electronic supplementary material:**

The online version of this article (doi:10.1186/s12881-016-0340-0) contains supplementary material, which is available to authorized users.

## Background

Chromosome microarray analysis of subjects with NDD has uncovered a large number of rare copy number variations (CNVs); nevertheless, some pathogenic and putatively pathogenic CNVs detected in patients cannot completely explain complex patient phenotypes, particularly when an unaffected parent carries the same submicroscopic imbalance. One example of a susceptibility locus for NDD is the 16p11.2 region with ~600 kb deletions and duplications observed in ~1 % of autism and 1.5 % of children diagnosed with significant developmental or language delays compared to 0.04–0.07 % amongst control populations [1, 2]. Carriers of 16p11.2 CNV manifest a broad spectrum of neurocognitive phenotypes, ranging from ID [1, 3, 4], autism spectrum disorder (ASD) [5, 6], schizophrenia [7], congenital anomalies [4, 8] to individuals without a specific phenotype [3, 4, 8]. There is familial coincidence of both phenotypically affected and unaffected carriers in some families [1, 7, 8]. The estimated penetrance of 16p11.2 deletion and duplication are 46.8 % and 27.2 %, respectively [2]. Multiple studies demonstrated the power of WES to find the genetic etiology of clinical variability among such patients. WES helped to discover that the presence of variants on the non-CNV containing homolog chromosome may unmask biallelic mutations in an autosomal recessive condition [9, 10], or that damaging variants in other parts of the genome may contribute to such variable expressivity [11]. The results of these studies suggest that inconsistent phenotypes in patients with known pathogenic CNVs or with CNVs inherited from an unaffected parent may indicate the co-occurrence of secondary genomic events elsewhere in the genome.

In this study, we report that pathogenic variants of *VPS13B* located at chromosome 8 in a boy with NDD carrying a familial dup16p11.2 contribute to the clinical variability in this family.

## Case presentation

**Fig. 1:**
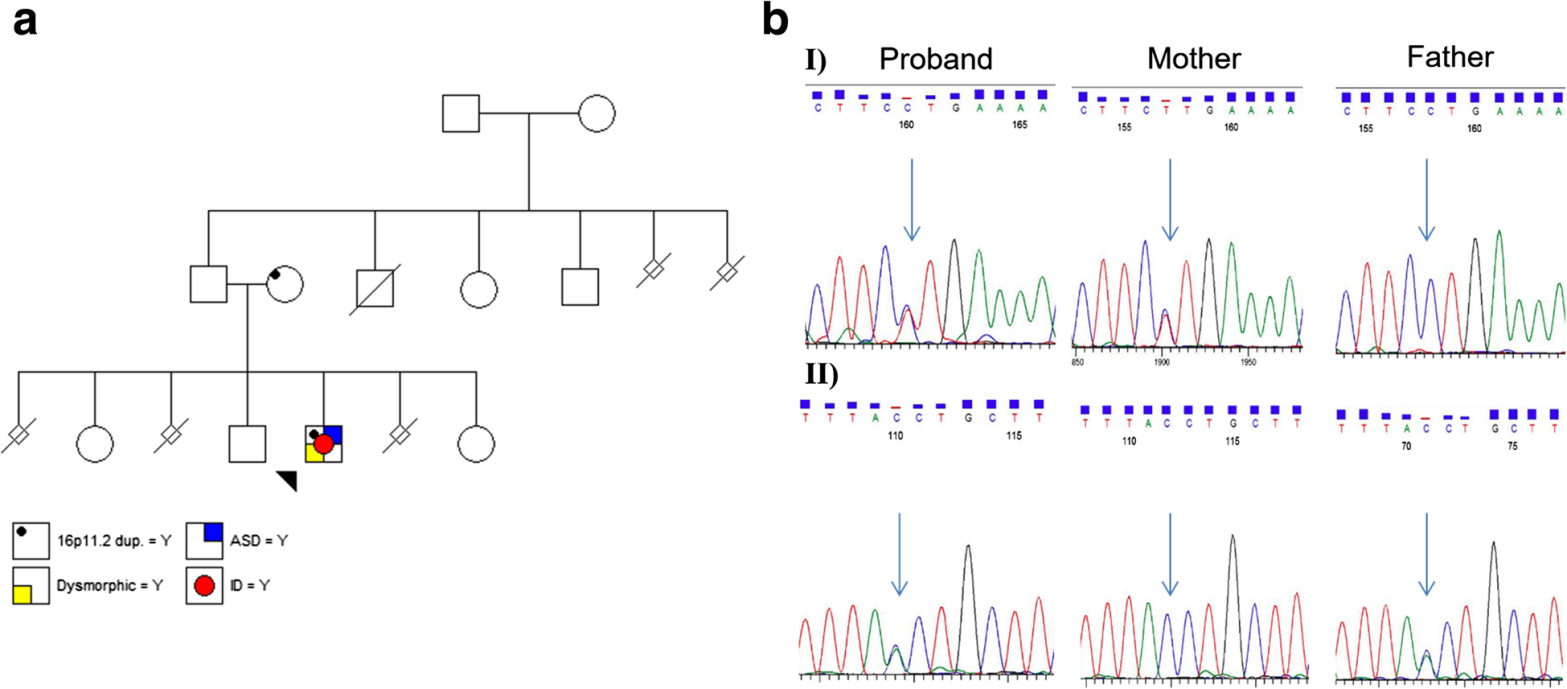
**a** Family pedigree. **b** Sanger sequencing analysis of *VPS13B* variants. I) Proband and his mother are carriers of splicing mutation of c.1426-1G > A. II) Proband and his father are carriers of splicing mutation of c.4157 + 1G > T (sequences of reverse strands are shown)

The proband is an 11 year old boy introduced to our clinic with global developmental delay and verbal apraxia at the age of four. He is the third of four-children of non-consanguineous parents of Chinese descent. His mother and his paternal grand-mother have a history of recurrent spontaneous pregnancy losses with unknown cause. His parents and three siblings are apparently healthy (Fig. 1a). The proband was born after 39 weeks of uneventful pregnancy via caesarean section for fetal distress with Apgar scores of 8 and 9 at one and five minutes after birth, respectively. His birth weight was 2175 gram (<3^rd^ percentile (%ile)), length was 47 cm (10^th^ %ile) and occipito-frontal circumference (OFC) was 34 cm (25^th^ %ile). The patient exhibited feeding difficulty, low muscle tone, bilateral ptosis, club foot, bilateral undescended testes, and flexion contracture of hand and wrist. The proband’s laboratory diagnostic workup was normal and included routine karyotype, subtelomeric FISH, fragile X, biochemical assessment, cranial MRI and CT scan. Affymetrix Genome-Wide Human SNP Array 6.0 revealed a 709.2 kb duplication of 16p11.2 (29,425,199–30,134,432) in the proband, confirmed by FISH and parental studies indicating maternal inheritance. The proband’s siblings were not tested for dup16p11.2 per the family’s request.

We examined the mother who is a carrier of dup16p11.2 for the possibility that apparently healthy carrier parents might have some unnoticed clinical features, and for the presence of phenotypic commonality with his child. She showed no sign of ID, ASD, psychiatric disorder (anxiety, depression, obsessive-compulsive disorders (OCD)), underweight or microcephaly. She was also negative for history of other dup16p11.2 features including epilepsy, speech and motor delay, and congenital anomalies.

### Genetic testing

DNA samples of family trios were sent to PerkinElmer Company for exome enrichment using the TruSeq Exome Enrichment Kit (Agilent v5 + UTR), followed by paired-end sequencing (Illumina HiSeq 2000, read length of 100 bp). Using Golden Helix (GH) software (SNP & Variation Suite 7.7.8), the WES data from a single VCF file for sequenced family members was analyzed (Additional file 1: Figure S1). Two novel splicing mutations of *VPS13B* (8q22.2) with compound heterozygous inheritance were identified in the proband and subsequently confirmed by Sanger sequencing (Fig. 1b). A sequence variant of c.1426-1G > A located in the acceptor splice site of intron 10 was identified in Proband A and his mother. The second variant, a nucleotide change of G > T at c.4157 + 1 situated in the donor site of intron 27, was inherited from his father. Mutations and/or CNVs in the *VPS13B* gene lead to a rare autosomal recessive condition called Cohen syndrome (CS) [12].

**Fig. 2:**
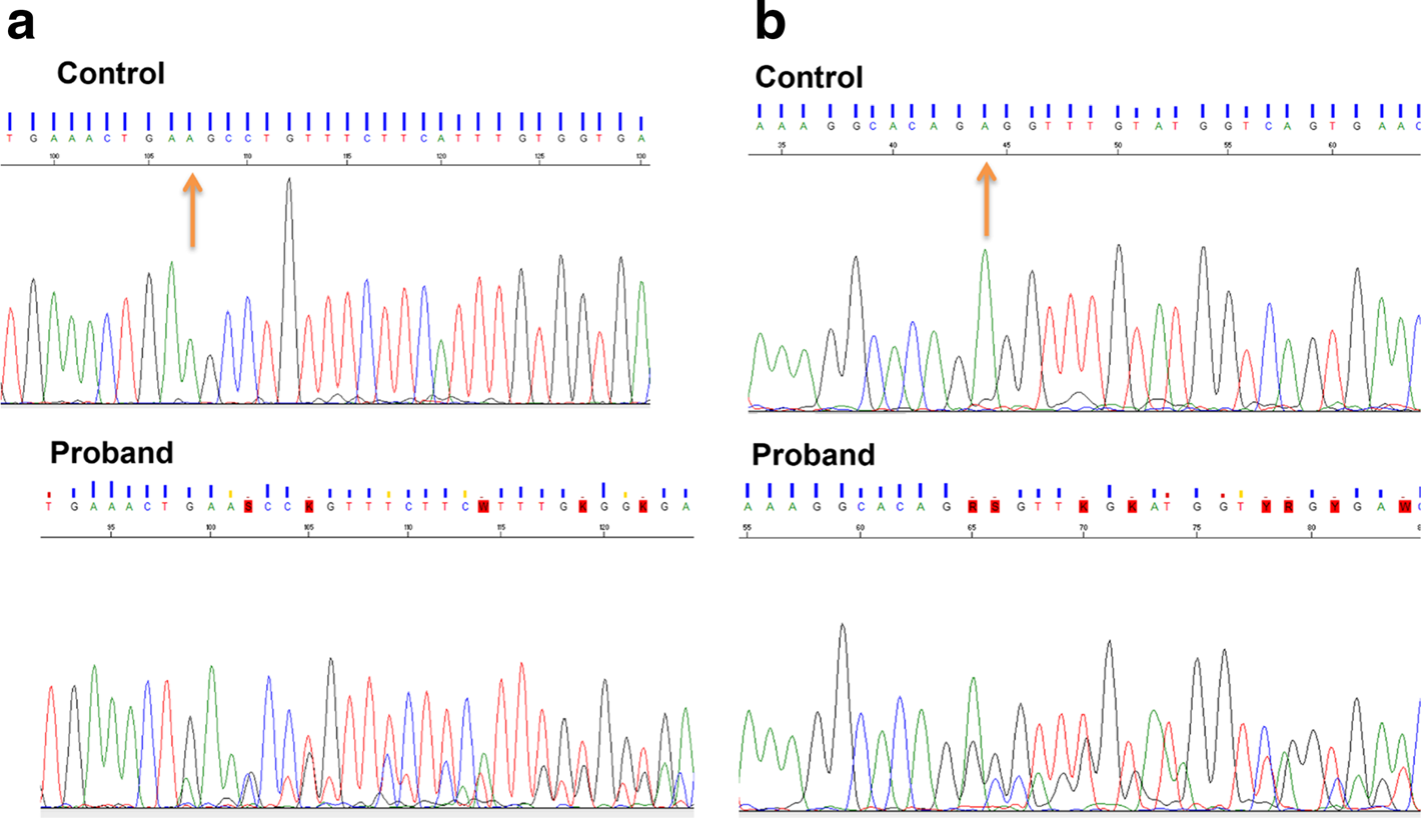
Sanger sequencing of RT-PCR products of proband and control, using primers covering exons 9–12 and 26–29 of *VPS13B*. **a** The variant of c.1426-1G > A disrupted the following sequences and caused frameshift in the proband. The orange arrow shows the first bp of exon 11 in the normal control. **b** The variant of 4157 + 1G > T disrupted following sequences, and caused frameshift in the proband. The orange arrow shows the first bp of exon 27 in the normal control

**Fig. 3:**
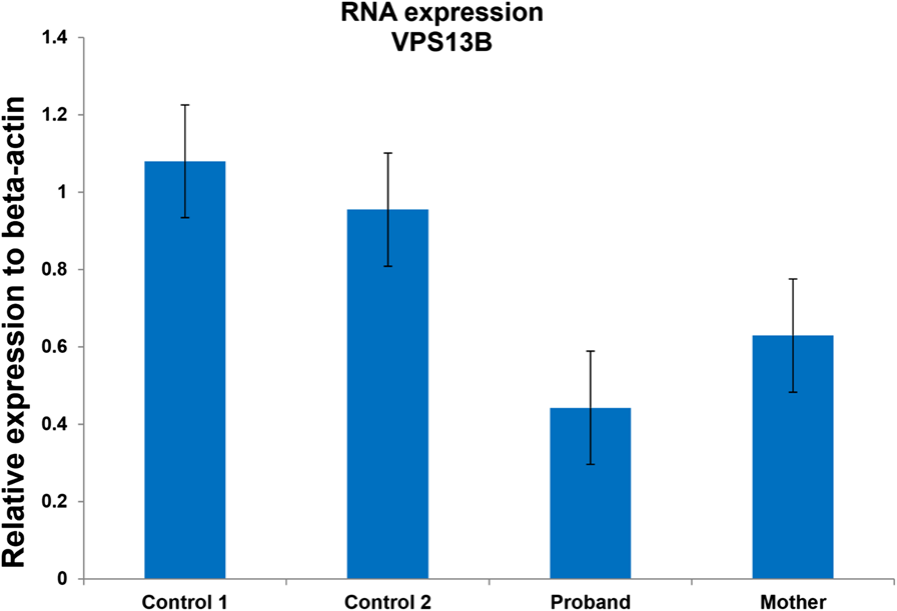
Expression study of *VPS13B* gene. The mean RNA expression of *VPS13B* calculated from three different time-series of RNA extraction in the proband, his mother and two normal controls. The relative expression of *VPS13B* is <0.5 fold in the proband and >0.6 fold in his mother. Error bars indicate standard errors from three replicates

Functional prediction tools used for WES data analysis anticipate the effect of non-synonymous variants (coding region). However, both variants of *VPS13B* are located at canonical splice sites. ALAMUT software predicted that two intronic variants of *VPS13B* would result in skipping of the exon 11 and 27. To confirm this prediction, we performed PCR on cDNA samples of proband and a control using two separate sets of primers covering exons 9–12 and 26–29 of *VPS13B,* followed by Sanger sequencing of the PCR products. This confirmed that both variants abolish the canonical splice sites and create aberrant RNA sequences (Fig. 2). Real-time quantitative PCR (qPCR) for *VPS13B* demonstrated reduced expression in the proband compared to two controls (Additional file 1: Material and methods). The mother also showed reduced RNA expression compared to one control (Fig. 3). Other family members were not available for *VPS13B* or dup16p11.2 testing.

The *VPS13B* gene, also known as *COH1* (OMIM: 607817), is approximately 864 kb in length and located on chromosome 8q22.2. It consists of 62 exons encoding a transmembrane protein of 4022 amino acids [12]. VPS13B is a peripheral membrane protein that is required for function, orientation and structural integrity of the Golgi apparatus and thus plays a role in vesicle-mediated sorting and intracellular protein transport [13, 14]. Homozygous or compound heterozygous mutations/CNVs of *VPS13B* cause CS [12]. ﻿

Intronic point mutations within donor and acceptor sites at mRNA splice junctions typically cause mRNA mis-splicing, leading to subsequent nonsense-mediated mRNA decay (NMD), and altered protein with effect on the clinical phenotype [15]. Indeed, Sanger sequencing of RT-PCR product corresponding to each specific *VPS13B* variant demonstrated that both variants create aberrant RNA sequences and frameshift and thus probably lead to NMD. Moreover, the RNA expression level of *VPS13B* in the proband was significantly reduced compared to two controls. *VPS13B* expression in his mother was intermediate between the proband and one control, suggesting that partial loss-of-function in carriers of autosomal recessive disorders is not sufficient to produce a complete disease phenotype.

Absence of dup16p11.2 -related phenotype in the mother, presence of some CS features in the proband, and the discovery of pathogenic *VPS13B* mutations warranted re-evaluation of our patient at 10 years of age. CS has a broad clinical phenotype spectrum including ID, microcephaly, hypotonia, dysmorphic facial features, truncal obesity, slender extremities, joint hypermobility, myopia, retinal dystrophy, intermittent isolated neutropenia, and happy personality. Neutropenia is characterized as a neutrophil count of <1.5 × 10^9^/L in children and <1.8 × 10^9^/L in adults [16]. The facial gestalt includes down-slanting palpebral fissures, wave-shaped eyelids, thick eyebrows and eyelashes, low hairline, prominent and beak-shaped nose, malar hypoplasia, short philtrum, high-arched palate, maxillary prognathia and prominent central incisors [17–19]. Patients with CS grimace when they are asked to smile [12, 20]. Other signs and symptoms include short stature and scoliosis [12, 20]. In addition, individuals with CS have high rates of ASD or autistic features [21]. The estimated prevalence of CS is 1:105,000 [22], however, its frequency may be considerably higher due to the fact that patients are often not diagnosed until they reach their teenage or adult years. The early diagnosis of CS is challenging because facial features are less noticeable in pre-school age, truncal obesity may evolve in late-childhood, neutropenia is rarely identified due to its intermittent pattern and absence of clinical consequences, and diagnosis of retinal dystrophy usually occurs in later childhood [16, 17, 20].

Reverse phenotyping of our patient at 10 years of age unequivocally confirmed a pattern of features consistent with CS (Table 1). Table 1 shows the presence or absence of clinical features observed in our proband relative to patients with CS [17, 18, 20–24], or dup16p11.2 [4, 8, 25–27].

**Table 1:** Clinical phenotypes of proband, and their presence/absence among reported cases of CS and dup16p11.2

		Reported findings in patients	
	Proband’s clinical findings	Cohen syndrome	dup16p11.2
Pregnancy/birth	Reduced fetal activity	+	-
	Low birth weight	+	-
	Feeding difficulty	+	-
Neurocognitive	DD/ID^ab^	+	+
	ASD^b^	+	+
	Happy/friendly disposition^a^	+	-
	Hypotonia^b^	+	+
	Verbal apraxia	+	-
	Motor delay^b^	+	+
	Poor motor coordination	+	+
	Brisk reflexes	+	-
Build/stature	Underweight^b^ (≤4y/o)	-	+
	Short stature	+	+
	Truncal obesity^a^ (childhood)	+	-
Cranium/hair	Microcephaly^a^ ^b^ (postnatal)	+	+
	Flat occiput	-	-
	Double hair whorls	-	-
	Low hairline^a^ (anterior)	+	+
	Thick hair^a^	+	-
Forehead/face/nose	Narrow forehead	+	-
	Micrognathia/mild retrognathia	+	+
	Malar hypoplasia	+	-
	Depressed nasal root	-	-
	Short triangular nose	-	-
Mouth/oral region	Small mouth	+	-
	Thick upper lip	+	-
	Short/smooth philtrum^a^	+	+
	High-arched palate	+	+
	Thickened alveolar ridges	-	-
	Prominent upper central incisors	+	-
Eye/eye globe/vision	Hypertelorism	+	+
	Ptosis (bilateral)	+	-
	Blepharophimosis	-	-
	Wave shaped eyelids^a^	+	-
	Thick eyebrow^a^	+	-
	Long/thick eyelashes^a^	+	-
	Myopia^a^	+	+
	Diffuse retinal dystrophy^a^	+	-
Ears/hearing	Large ears	+	+
	Posteriorly rotated ears	+	+
	Auricular pits	+	+
	Hearing loss (unilateral sensorineural)	+	-
Abdomen/thorax	Diastasis recti	-	-
	Hypoplastic nipples	-	-
Extremities/musculoskeletal	Slender extremities/tapered fingers^a^	+	+
	Joint hypermobility^a^	+	+
	Club foot (bilateral)	+	-
	Sandal gap	+	+
	Scoliosis	+	+
Genitalia/urinary tract	Hypospadias	-	+
	Cryptorchidism	+	+
Haematology/Immunology	Chronic anemia	+	-
	Recurrent infection (UTI)	+	-
	Intermittent neutropenia^a^	+	-

^a^Diagnostic criteria for CS

^b^Most observed findings among patients with dup16p11.2

Being underweight is a known feature of dup 16p11.2. Although the proband was underweight at birth, his weight changed with age to the 5–10^th^ %ile at the age of 10. He also developed truncal obesity with slender extremities, mild scoliosis, and evolving facial gestalt consistent with CS. Similar to the report by El Chehadeh-Djebbar et al. [17], our study suggests that some CS features are age-dependent and evolve later in childhood (Table 2).

**Table 2:** Evolving clinical features of proband

Evolving features	4y/o	10y/o
Weight	<3^rd^ %ile	5–10^th^ %ile
Oval face	No	Yes
Truncal obesity	No	Yes
Down-slanting, wavy palpebral fissures	No	Yes
Short and smooth philtrum	No	Yes
Long slender distal extremities/fingers	No	Yes
Spine abnormality	No	Yes

## Conclusion

Inherited dup16p11.2 by itself cannot explain the variable expressivity among NDD patients when their carrier parents are unaffected. We utilized WES in a family with a child presenting with NDD carrying dup16p11.2 inherited from his unaffected mother, and searched for sequence changes that could explain this clinical variability. We discovered that compound heterozygous variants of *VPS13B* contribute to the proband’s phenotypic features. The new CS diagnosis helps in screening and earlier management of scoliosis, periodontal disease and tooth loss, early cataract, vision loss, and premature aging [24] in the proband; and provides more informed genetic counselling for the family.

Our study suggests that NDD patients with dup16p11.2 may show additional pathogenic SNVs in their genome, which significantly influence phenotype heterogeneity and the genetic counselling of families with putatively pathogenic CNVs showing variable expressivity and incomplete penetrance. Genomic microarray is a valuable first-tier test for the postnatal evaluation of individuals with NDD including ID, ASD, and/or multiple congenital anomalies. However, coupling of microarray with WES or whole genome data analyses will facilitate a more comprehensive and accurate analysis of genetic causes of NDD, heighten understanding of the etiology of variable expressivity among NDD patients, and optimize clinically-informed and effective genetic counselling and personalized management options.

## Additional file

Additional file 1: Figure S1: Filtering strategies used for analysis of WES data; Additional material and methods. (DOCX 83 kb)

## Abbreviations

%ile: Percentile
ASD: Autism spectrum disorder
CNV: Copy number variation
CS: Cohen syndrome
Dup: Microduplication
ID: Intellectual disability
NDD: Neurodevelopmental disorders
NMD: Nonsense-mediated mRNA decay
OCD: Obsessive-compulsive disorders
OFC: Occipito-frontal circumference
qPCR: Real-time quantitative PCR
WES: Whole exome sequencing
