# Supplementary material for: Exome sequencing identifies pathogenic variants of VPS13B in a patient with familial 16p11.2 duplication
Source: BMC Med Genet. 2016 Nov 10;17:78. doi: 10.1186/s12881-016-0340-0 (PMC5105257; doi:10.1186/s12881-016-0340-0)

# Material and Methods

## Whole exome sequencing

Genomic DNAs of the family trio were extracted from the whole blood using the QIAamp DNA blood Mini Kit (QIAGEN), and then were enriched using the TruSeq Exome Enrichment Kit (Agilent v5+UTR), followed by paired-end sequencing (Illumina HiSeq 2000, read length of 100 bp). Reads were mapped using BWA against the genomic reference sequence for Homo sapiens (Build 37). Using Golden Helix software, the WES data from a single VCF file for all sequenced family members was analysed. The filtering process is shown in Figure S1.

## Sanger sequencing

Primers were designed using the “Primer3 software”. After PCR optimization in controls, PCR on genomic DNAs of the specific family, followed by PCR clean-up were performed. Next, direct PCR product sequencing was conducted using Bigdye terminator V3.1 cycle sequencing kits, and subsequently analysed on ABI 3130XL genetic analyzer.

## Real time quantitative PCR (qPCR)

Three different replicates of RNA were extracted from lymphoblastoid cell lines (LCLs) using RNeasy Mini Kit (QIAGEN). cDNAs were made using EasyScript™ cDNA Synthesis Kit (Applied Biological Material Inc.). qPCR was conducted on the Applied Biosystems StepOne Plus^TM^ Real-time PCR system using TaqMan Gene Expression Assay for the *VPS13B* genes (Hs00215450). Quantification of the expression level of *VPS13B* was performed in comparison to beta-actin. The mean expression of three replicates was calculated for each studied individual separately.

**Figure S1:** **Filtering strategies used for analysis of WES data**

Abbreviation: SNV: single nucleotide variation, NHLBI: National Heart, Lung and Blood Institute, MAF: minor allele frequency.


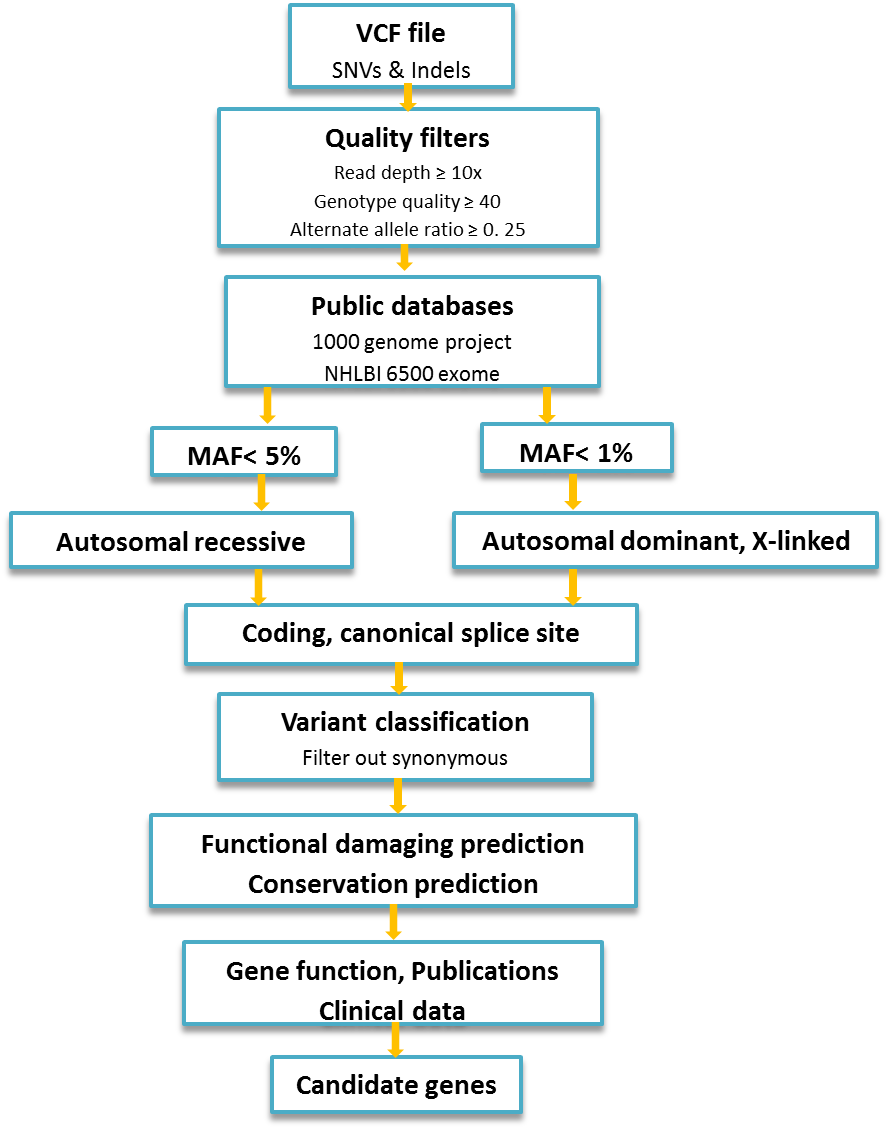

Supplement: Additional file 1: — Figure S1: Filtering strategies used for analysis of WES data; Additional material and methods. (DOCX 83 kb) [file 12881_2016_340_MOESM1_ESM.docx]
